# Development of a Two-Layer Porous Scaffold Based on Porcine Nasal Septal Cartilage for Orthopedics

**DOI:** 10.17691/stm2021.13.4.05

**Published:** 2021-08-28

**Authors:** N.Yu. Ignatieva, O.L. Zakharkina, E.A. Sergeeva, N.B. Serezhnikova, A.L. Faizullin, A.B. Shekhter

**Affiliations:** Associate Professor, Chemical Faculty; Lomonosov Moscow State University, 1, bld. 3 Leninskiye Gory, Moscow, 119991, Russia; Researcher; Institute of Photonic Technologies of Federal Scientific Research Center “Crystallography and Photonics” of the Russian Academy of Sciences, 2 Pionerskaya St., Moscow, Troitsk, 108840, Russia; Senior Researcher; Federal Research Center Institute of Applied Physics of the Russian Academy of Sciences, 46 Ulyanova St., Nizhny Novgorod, 603950, Russia; Senior Researcher; I.M. Sechenov First Moscow State Medical University (Sechenov University), 8/2 Malaya Trubetskaya St., Moscow, 119991, Russia; Junior Researcher; I.M. Sechenov First Moscow State Medical University (Sechenov University), 8/2 Malaya Trubetskaya St., Moscow, 119991, Russia; Chief Researcher, Laboratory of Experimental Morphology and Biobank, Institute for Regenerative Medicine; I.M. Sechenov First Moscow State Medical University (Sechenov University), 8/2 Malaya Trubetskaya St., Moscow, 119991, Russia

**Keywords:** porous scaffold, cartilage tissue, chemical modification, glyceraldehyde, ribose

## Abstract

**The aim of the study** was to design a construct based on a nasal septal cartilage plate providing required cell differentiation in different layers to replace a deep osteochondral defect and develop an algorithm of chemical and physical effect sequence to create non-immunogenic two-layer porous structure with requisite elasto-mechanical properties.

**Materials and Methods:**

The plates derived from porcine nasal septal hyaline cartilage covered by perichondrium were multi-stage treated including freezing, equilibrating in a hypotonic saline solution (type I specimens); trypsinization, point IR-laser effect, re-trypsinization (type II specimens); a stabilizing effect of crosslinking agents — glyceraldehyde/ribose in an acidic medium — washing (type III specimens).

For all type specimens:

1. there were established stability parameters (collagen denaturation temperature using a thermal analysis; and Young’s modulus using a mechanical analysis);
2. there were determined morphological characteristics using light and polarization microscopy with classical staining and nonlinear optical microscopy in second-harmonic generation mode.

**Results:**

Thermal, mechanical, and morphological properties in type I specimens slightly differed from those of the initial nasoseptal system. A considerable part of cells had destroyed membranes.

In type II specimens, thermal stability of collagen frame was significantly lower; Young’s modulus decreased more than fourfold compared to type I specimens. Collagen structure of hyaline cartilage appeared to be disarranged, although the morphological differences of the hyaline part and perichondrium preserved. The construct matrix was almost completely decellularized. Successive exposure to laser radiation and trypsin resulted in the formation of partial holes in the matrix, ~100 μm in diameter.

In type III specimens, both the thermal stability of the collagen frame and Young’s modulus (E) increased. Glyceraldehyde was more effective than ribose, E having reached the value typical for intact hyaline cartilage. Collagen fibers in type III specimens were thicker than in type I and II specimens. The morphological differences of the hyaline part and perichondrium and partial holes were preserved.

**Conclusion:**

Due to sequential treatment by salts, trypsin, IR-laser radiation, and nontoxic crosslinking agents, nasal septal cartilage plate forms porous acellular construction consisting of two layers formed by type I (from perichondrium) and type II (from hyaline part) collagen fibers. In the present construction, stability, mechanical properties, and size of the partial holes can be assigned for cell colonization. It enables to use the construction to replace articular cartilage defects.

## Introduction

Deep osteochondral defects due to injuries and osteochondrosis present one of the most common problems in locomotion pathology therapy. Over the past two decades, there has been introduced a novel approach to tissue regeneration determining implantation of tissue-engineering constructs [1].

A construct used for an articular cartilage should meet certain requirements [2–5]: 1) to be non-immunogenic, biodegradable, and biocompatible; 2) to be able to be extensionally cell-colonized; 3) to preserve the differentiation of chondrocytes or to differentiate in chondrocytes when inoculating scaffold by stem cells; 4) to comply with osteochondral structure by its elasto-mechanical properties and heterogeneous multi-layer structure.

It is likely that an ideal construct is to be considered the one made from self-tissue (an autograft), however, the approach has a certain restriction: a replacement of a deep osteochondral defect results in defects forming elsewhere. A promising basis for cartilaginous tissue scaffolds is decellularized matrix of animal cartilaginous tissue proper — xenograft [3–13], which complies with requirements 1 and 3. Decellularization is a multi-stage process of combined chemomechanical treatment [4], after which cartilaginous tissue preparation fails to meet requirement 4. It should be noted that decellularization without mechanical grinding is rather labor-consuming process; moreover, the remaining collagen matrix appears to be too dense to ensure compliance with requirement 2 [7]. To create material with directed collagen fibers and pores from cartilage powder needs, as a rule, special press molds with programmable temperature change [8, 10, 11]. Another challenging technique to create channels in collagen matrix was suggested in the study [9], where the needles, 400 μm in diameter, were used.

One of the most complicated problems in creating a construct replacing an osteochondral defect is related to multilayered damaged tissue of an articular cartilage; its upper part is a hyaline cartilage formed by type II collagen, and lower part is a subchondral bone tissue formed on the basis of type I collagen. Different variants to fabricate a single stable construct satisfying requirement 4 have been suggested; however, most methods are highly technical; among these are space-division transgene immobilization encoding different growth factors for chondrogenic or osteogenic stem cell differentiation [14], and mechanical combination of a decellularized ground cartilage and a mineral (or a polymer) subsystem [15–18].

As a primary part for scaffolds, we suggest using a nasal septum part including the hyaline cartilage (type II collagen) and perichondrium (type I collagen). After decellularization and forming pores, and the recovery of collagen framework mechanical properties due to the formation of additional transverse bonds, it is possible to create a supporting construct to replace osteochondral defects meeting requirements 1–4. It should be noted that partial holes ~100 μm in size are considered optimal for free colonization with chondrocytes [9].

**The aim of the study** was to design a construct based on a nasal septal cartilage plate providing required cell differentiation in different layers to replace a deep osteochondral defect and develop an algorithm of chemical and physical effect sequence to create non-immunogenic two-layer porous structure with requisite elasto-mechanical properties.

## Materials and Methods

As an initial system, we used the plates, 7×10 mm in size, and 2 mm thick, cut out of the porcine nasal septum (not later than 12 h after sacrifice). The plates were covered by a thin perichondral layer, the bottom was the hyaline cartilage part.

The preliminary treatment was the plates freezing within 6 days at –18°С and subsequent equilibrating for 24 h at 37°С in a hypotonic saline solution (0.1 M NaCl with added 0.01 М Tris-HCl and 0.005 M MgCl_2_).

Enzymatic treatment consisted in incubating the plates for 24 h at 37°С in NaCl solution, 0.15 М, containing trypsin, 1 mg/ml. The treatment was performed twice: before and after IR-laser irradiation.

The crosslinking agent treatment involved incubating the plates within a specified period of time (24, 48, or 120 h) at 24°С in NaCl solution, 0.15 М, with HCl added up to the concentration reaching 0.32 mM (pН 3.5) containing a crosslinking agent. As a crosslinking agent, we used glyceraldehyde, which formed *in situ* from its precursor — diethyl acetal of glyceraldehyde in an acidic medium. The precursor was added in the solution so that the concentration was 0.02 М. Another crosslinking agent — ribose (concentration 0.02 М) — was also tested. Such treatments were conducted after IR-laser exposure.

After each treatment (enzymatic treatment and crosslinking agent treatment), the specimens were washed in 300 ml NaCl solution 0.15 М using a filter funnel under vacuum of a water-jet pump. Washing time of each specimen was about an hour.

To form partial holes in the material, we used erbium-doped fiber laser radiation, 1.56 μm wavelength. Radiation was delivered through a quartz optical fiber, the core diameter being 400 μm. There was a contact action, the field of incidence diameter corresponded to the fiber diameter. The construct plate was exposed to radiation bilaterally, by laser spot rows; the distance between the rows and two adjacent spots was about 2 mm. At such distance between the spots, the matrix change in each exposure area was due to a direct laser radiation effect at the site fibers contact with the material and does not depend on radiation at adjacent points.

Collagen thermal stability of the specimens was measured by a differential scanning calorimeter (model DSC 204 F1; Netzsch, Germany). Specimens weighing 5–8 mg were hermetically sealed in standard aluminum dishes, their volume being 20 μl. Initial and end temperatures were 20 and 90°С, respectively; the heating rate was 10 K/min.

Mechanical testing was conducted using a universal desk testing machine EZ Test (model EZ-SX; Shimadzu, Japan) at room temperature, under the conditions of uniaxial compression in the direction perpendicular to the plate surface. Vertical load (up to 20 Н) was determined using a force-monitoring system, the rate of displacement of the compressing plate was 0.5 μm/s. Using automatically recorded data “load–displacement” we derived the relation “stress (σ) – strain (ε)”, which was further approximated using the exponential function σ=*A*·exp(*B*ε) with two adjustable parameters (*A*, *B*). The first-order derivative value of an approximate function, if ε=10%, was taken as Young’s modulus (E).

For morphological studies, we fixed the specimens in 10% neutral formalin, and prepared paraffin sections, 4 and 16 μm thick. Thinner sections stained by hematoxylin and eosin and picrosirius red according to a standard technique were studied using a universal optic microscope Leica DM4000 B LED (Leica Microsystems, Switzerland) in the modes of light, phase contrast, and polarization microscopy. Microphotographs were taken using a digital video camera Leica DFC7000 T with LAS V4.8 software. The sections, 16 μm thick, were visualized in the second-harmonic generation (SHG) mode and studied using a laser scanning microscope LSM 510 META (Carl Zeiss, Germany). The excitation was carried out by pulse femtosecond radiation of Ti:Sa-laser (Mai Tai HP; Spectra-Physics, USA), wavelength 800 nm, pulse duration 100 fs, and pulse repetition rate of 80 MHz. SHG signal was separated using a dichroic filter of visible radiation (HFT KP 650; Carl Zeiss) and a narrow-band filter (400/10 nm).

**Figure 1:**
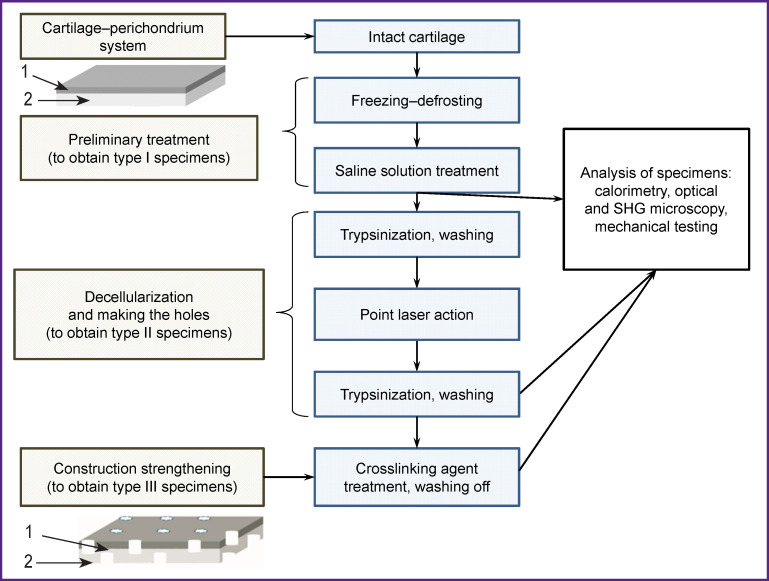
Block diagram of the experiment: *1* — perichondrium; *2* — hyaline cartilage

Figure 1 shows the experimental design including the preparation of specimens and the method used to study them.

## Results

**Figure 2:**
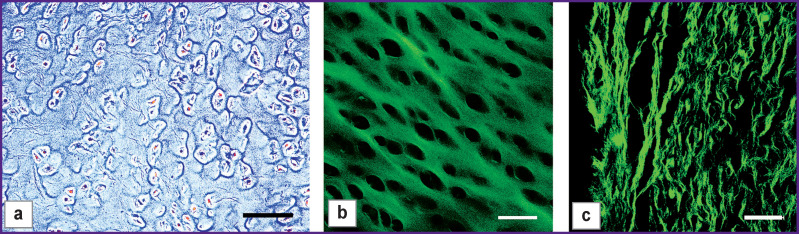
Microphotographs of type I specimen sections: (а) hyaline cartilage, hematoxylin and eosin staining, phase contrast microscopy; (b) hyaline cartilage; (c) perichondrium; SHG images. Bar — 50 μm

A morphological examination of the specimens after the preliminary treatment (type I) showed most chondrocytes to be characterized by cell nuclei deformity and karyorrhexis; collagen matrix structure was preserved both in the hyaline part and in the perichondrium (Figure 2).

A thermal analysis confirmed that collagen in both parts preserved thermal stability: in the hyaline part collagen does not denatures up to 95°С, and in the perichondrium, denaturation occurs at 63.5±0.5°С.

Mechanical properties of type I specimens were no different from intact ones: Young’s modulus (Е) was 0.65±0.15 MPa that was close to Е=0.70±0.10 MPa typical for intact specimens.

To form partial holes, type I specimens at the first stage were enzymatically treated, proteolysis products including glycosaminoglycan chains were washed off. After such procedure, thermal stability of perichondral collagen was unchanged; however, collagen in the hyaline part was able to denature completely at 61.8±0.5°С.

Local denaturation requires, on the one hand, laser heating time to be significantly lesser than thermal relaxation time (~0.1 s in radial direction [19]), and, on the other hand, the temperature in the object exposed to radiation to be not less than 61°С. Therefore, there were chosen the pulse durations: 50 and 100 ms, laser radiation power was 2–4 W [20]. After point laser treatment, the specimens were re-trypsinized and washed off from proteolysis products.

Geometric characteristics of the partial holes were measured using a laboratory microscope with the accuracy of about 10 μm. It turned out that at 2 W power and pulse duration 100 ms, or 3 W power and pulse duration 50 s, holes were formed, from 90 to 120 μm in diameter, that was close to the target size. In this way, we obtained type II specimens.

**Figure 3:**
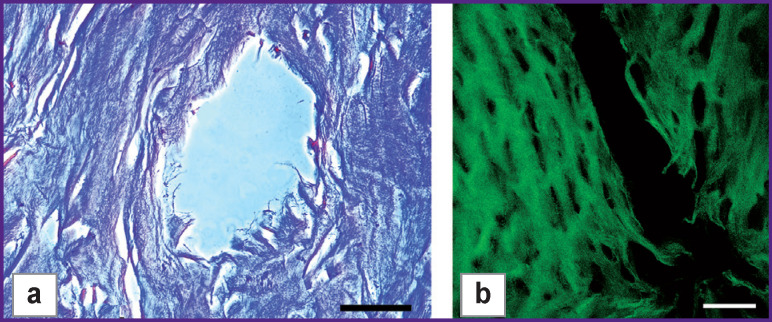
Laser openings on transverse (а) and longitudinal (b) sections of type II specimens: (а) hematoxylin and eosin staining, phase contrast microscopy; (b) SHG image. Bar — 50 μm

The microphotographs of longitudinal and transverse sections of type II specimens show the matrix actually has partial holes formed, their diameter being close to target values: ~100 μm (Figure 3).

**Figure 4:**
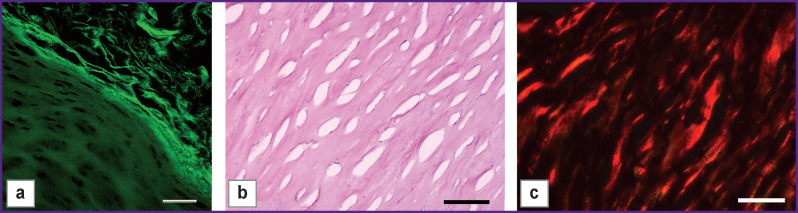
Microphotographs of type II specimen sections: (а) SHG image; (b) hematoxylin and eosin staining; (c) picrosirius red staining, polarization microscopy. Bar — 50 μm

Type II specimens were found to have significant morphological changes compared to type I specimens. The microphotographs show most lacunae be empty, without cell nuclei (karyolysis), only some of them having nuclear fragments (karyorrhexis). Collagen fibers were found in the perichondrium, as well as in the hyaline part; however, the hyaline fibrillar structure was damaged (Figure 4). In normally homogeneous matrix, thin fibrous structure appears, while collagen anisotropy in polarization microscopy occurs not everywhere.

According to the thermal analysis, perichondral collagen stability in type II specimens changed slightly compared to the hyaline part collagen, where denaturation was within the range 48–61°C, with maximum peak at endotherm ~55°C. Young’s modulus decreased up to 0.15±0.10 MPa.

**Figure 5:**
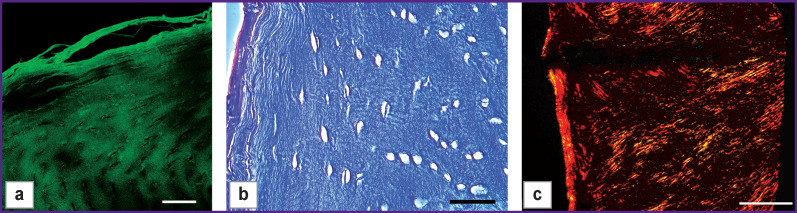
Microphotographs of type III specimen sections after exposure to glyceraldehyde within 48 h: (а) SHG image; (b) hematoxylin and eosin staining, phase contrast microscopy; (c) picrosirius red staining, polarization microscopy. Bar — 50 μm (а), (b) and 500 μm (c)

Collagen framework was strengthened due to the treatment with crosslinking agents (glyceraldehyde and ribose) resulting in type III specimens, which were characterized by almost total absence of cellular nuclei in preserved collagen framework and laser partial holes (Figure 5). Collagen matrix architectonics of the hyaline part was significantly changed: the structure became denser, the fibers arranging in bundles. The perichondral fibers preserved their orientation, which was parallel to the surface.

Collagen thermal stability in type III specimens increased. Specimen crosslinking time increase resulted in denaturation time growth. Glyceraldehyde as a crosslinking agent appeared to be more active than ribose. Thermal analysis findings of type III specimens are presented in the Table.

**Table:** Thermal and mechanical characteristics of the specimens when preparing scaffolds

Specimen type	Denaturation temperature of the hyaline part (°C)	Young’s modulus (MPa)
I	>95	0.65±0.15
II	55±2	0.15±0.10
III GA-24	62±1	0.65±0.10
III GA-48	64±1	0.7±0.1
III R-48	59±1	0.3±0.1
III R-120	62±1	0.6±0.2

Here: GA — glyceraldehyde; R — ribose; 24, 48, and 120 — exposure time of a crosslinking agent (h).

Mechanical stability in type III specimens increased, Young’s modulus turned out to be close to Е value for intact collagen tissue. Similar to denaturation temperature, glyceraldehyde treatment led to greater E increase compared to ribose, even in lesser exposure time. Crosslinking time increase using glyceraldehyde resulted in Е growth. Е values for type III specimens are also presented in the Table.

## Discussion

The preparatory treatment of cartilaginous plate had an effect, primarily, on cell condition. Indeed, freezing–defrosting and hypotonic solution treatment initiated cell membrane damage. Thus, the first destruction stage occurred in type I specimens. First trypsinization of type I specimens makes it possible to denature collagen of the hyaline cartilage part in IR-laser heating [21]. As a result, after laser irradiation and re-trypsinization, in its turn launching proteolysis of denatured collagen, partial holes form in preparations. Holes radius and length depend on laser action parameters. Wavelength of radiation (λ) determined radiation penetration depth; fiber diameter — a zone of treatment. Pulse duration (*t*) and power (*Р*) cause maximum temperature, and their variations enable to correct variation range in tissue [22]. To form holes, 100 μm in radius and ~1 mm long, we chose λ=1.56 μm and an appropriate fiber diameter for contact action. Other required characteristics (*Р* and *t*) were determined experimentally: preliminarily, we had assessed their range in previous studies [20]. Thus, changing IR-laser irradiation parameters it is possible to vary geometrical characteristics of the holes in the collagen frame within a rather wide range.

It is significant that double proteolytic enzymatic treatment leading to the destruction of all proteins (including intracellular ones), and double washing of proteolysis products results in almost complete matrix decellularization. Presumably, membrane destruction and DNA and RNA, which are freed from stabilizing protein membranes, contribute to cellular material removing. Unfortunately, collagen frame loses its stability including thermal and mechanical ones, due to the destruction of the proteoglycan aggregate subsystem [21, 23].

Nearly fivefold decrease of Young’s modulus in type II specimens puts in question their application to replace cartilaginous parts, which are subjected to compression when a joint works. To enhance stiffness and mechanical strength of collagen-based materials, there being successfully used crosslinking agent treatment [24–26]. The first agents were multifunctional aldehydes, in primary reaction forming the bonds between an aldol group and a free amino group of lysine residue included in a collagen polypeptide chain [24]. The increase in the number of covalent bonds between collagen molecules is likely to result in stiffness growth of the material consisting of single fibers [27]. The main requirements to crosslinking agents (in particular, aldehydes) are their sufficient chemical activity in addition reaction, on the one hand, and cytotoxicity absence — on the other hand. As crosslinking agents, we chose 2 aldehydes: more active glyceraldehyde with extremely low cytotoxicity, and much less active ribose, which is absolutely non-cytotoxic. Both agents exhibited their ability to stabilize the collagen frame of a construct (type III specimens of glyceraldehyde and type III specimens of ribose). Moreover, if reaction time increases, the bridging degree values (denaturation temperature and Е) grew, however, in compliance with expected activity. Thus, after 24 h of glyceraldehyde action, mechanical characteristics of the material were equal to those for the initial hyaline cartilage. Collagen became significantly more thermostable. As for active ribose, which is less chemically active, we managed to reach the target value after three-day reaction. Thus, the use of nontoxic crosslinking agents enables to obtain scaffolds based on an integrated system of collagen II and I fibers, primarily, divided in space, the mechanical characteristics of a construct can be varied in a wide range.

We carried out the reactions leading to crosslinking formed between macromolecules at рН 3.5, in contrast to the studies [25–28], which also used these crosslinking agents. It is impossible to use an acidic medium for *in vivo* crosslinking, however, for scaffold treatment, it is not a barrier, since excess acid can be neutralized by the base or washed off. On the other hand, the formation of the first covalent bond between aldehyde and lysine residue involves protonation stage and a reaction in an acidic medium proceeds considerably faster. Indeed, in a neutral medium, it is possible to achieve significant changes of stability characteristics under ribose action within 5–7 days [25–28]. In our case, the effect was achieved as early as in 3 days.

The essential result is the preservation of a single collagen frame and hyaline part represented by collagen II, and perichondrium chiefly consisting of collagen I. Such spatial division of different types of collagen in a uniform system can provide necessary differentiation in both osteogenic and chondrogenic parts.

## Conclusion

Due to sequential treatment by salts, trypsin, IR-laser radiation, and nontoxic crosslinking agents, nasal septal cartilage plate forms porous acellular construction consisting of two layers formed by type I (from perichondrium) and type II (from hyaline part) collagen fibers. In the present construction, stability, mechanical properties, and size of the cavities can be assigned for cell colonization. It enables to use the construction to replace articular cartilage defects.
